# Characterisation of Signalling by the Endogenous GPER1 (GPR30) Receptor in an Embryonic Mouse Hippocampal Cell Line (mHippoE-18)

**DOI:** 10.1371/journal.pone.0152138

**Published:** 2016-03-21

**Authors:** Nicholas J. Evans, Asha L. Bayliss, Vincenzina Reale, Peter D. Evans

**Affiliations:** The Signalling Laboratory, The Babraham Institute, Cambridge, CB22 3AT, United Kingdom; Universidad Miguel Hernández de Elche, SPAIN

## Abstract

Estrogen can modulate neuronal development and signalling by both genomic and non-genomic pathways. Many of its rapid, non-genomic effects on nervous tissue have been suggested to be mediated via the activation of the estrogen sensitive G-protein coupled receptor (GPER1 or GPR30). There has been much controversy over the cellular location, signalling properties and endogenous activators of GPER1. Here we describe the pharmacology and signalling properties of GPER1 in an immortalized embryonic hippocampal cell line, mHippoE-18. This cell line does not suffer from the inherent problems associated with the study of this receptor in native tissue or the problems associated with heterologously expression in clonal cell lines. In mHippoE-18 cells, 17β-Estradiol can mediate a dose-dependent rapid potentiation of forskolin-stimulated cyclic AMP levels but does not appear to activate the ERK1/2 pathway. The effect of 17β-Estradiol can be mimicked by the GPER1 agonist, G1, and also by tamoxifen and ICI 182,780 which activate GPER1 in a variety of other preparations. The response is not mimicked by the application of the classical estrogen receptor agonists, PPT, (an ERα agonist) or DPN, (an ERβ agonist), further suggesting that this effect of 17β-Estradiol is mediated through the activation of GPER1. However, after exposure of the cells to the GPER1 specific antagonists, G15 and G36, the stimulatory effects of the above agonists are replaced by dose-dependent inhibitions of forskolin-stimulated cyclic AMP levels. This inhibitory effect is mimicked by aldosterone in a dose-dependent way even in the absence of the GPER1 antagonists. The results are discussed in terms of possible “Biased Antagonism” whereby the antagonists change the conformation of the receptor resulting in changes in the agonist induced coupling of the receptor to different second messenger pathways.

## Introduction

The G-protein coupled receptor (GPCR) sensitive to estrogen (GPER1 or GPR30) appears to mediate many of the rapid, non-genomic actions of estrogen in a wide variety of tissues, including the brain and various cancer cell lines (see [1]). There has been considerable controversy regarding its cellular location, signalling pathways and even the nature of its endogenous agonists (see [1–3]). Although some initial studies suggested the receptor was expressed in the plasma membrane (see [4, 5]), other studies suggested the receptor was exclusively expressed in the endoplasmic reticulum and trans-Golgi network [6].Nevertheless, later studies have conclusively demonstrated that the receptor can be expressed in the plasma membrane [7] and that its plasma membrane localization can be enhanced and stabilized by an association with scaffolding proteins containing PDZ binding domains, such as post synaptic density protein 95 and synapse associated protein 97, as well as with a range of other proteins, including a range of other GPCRs [8–10]. However, rapid non-genomic responses to estrogens have been reported to be due to the activation of a range of additional plasma membrane located receptors in a wide variety of cell types in the nervous system (see [11, 12]). Thus, the classical estrogen receptors, ERα and ERβ, have been suggested to have a plasma membrane location in nervous tissue, where they can mediate some of the rapid non-genomic actions of estrogen (see [12]). These classic estrogen receptors may be located at the plasma membrane by coupling to other membrane receptors such as glutamate metabotropic receptors or by palmitoylation. In addition, a membrane bound estrogen receptor coupled to Gq proteins and blocked by STX (Gαq-mER or STX receptor) has been suggested to be responsible for the rapid estrogenic desensitization of μ-opioid and GABA_B_ receptors in proopiomelanocortin expressing neurons in the hypothalamus (see [13]). Further, cortical neurons, and neurons from many other regions of the brain, have been suggested to express an additional estrogen receptor, ERX, which can activate the MAPKinase cascade and is associated with caveolar-like microdomains (see [14]). However, the molecular identity of the latter two receptors remains unknown.

GPER1 has been reported to be able to couple to a wide range of signalling pathways both when expressed heterologously in clonal cell lines or homologously in a range of cancer cell lines and native tissues (see [1, 5]). Thus, GPER1 has been reported to mediate a Gs stimulation of cyclic AMP levels, a Gi/o mediated activation of extracellular signal-regulated kinase (ERK)1/2 via a complex pathway involving the trans-activation of epidermal growth factor receptor (EGFR), as well as activation of the phosphatidylinositol 3-kinase (PI3K) /Akt (also known as Protein Kinase B) pathway (see [2]). However, the identities of the pathways mediating many of the rapid, non-genomic actions of 17β-Estradiol in various tissues are not clear. There is also a current controversy over whether aldosterone can act as an endogenous activator of GPER1 in some tissues, particularly those from the cardiovascular system (see [3]).

Although a wide variety of studies have indicated a role for 17β-Estradiol activation of GPER1 in hippocampal tissue from the brain [8, 9, 15–18] the identification of the definitive molecular pathways activated by GPER1 is inherently difficult in intact brain tissue and in primary cultures of undefined isolated hippocampal neurons [19]. Thus, Gingerich et al., [19] have generated a number of immortalized cell lines from embryonic (E18) and adult derived hippocampal primary cell cultures using retroviral infection of SV40 –T antigen. The mHippoE-18 clonal embryonic line has a strong level of GPER1 expression combined with moderate expression of the classical estrogen receptors, ERα and ERβ. It thus represents a very useful tool in which to examine signalling by GPER1 in a natural cellular environment. The present study reports on the characterization of the pharmacology and signalling properties of endogenously expressed GPER1 in the mHippoE-18 clonal embryonic cell line.

## Materials and Methods

### Culture of mHippoE-18 cells

mHippoE-18 cells were obtained from VH Bio Ltd and maintained in culture as recommended by CELLutions Biosystems Inc., Burlington, Ontario, Canada. Briefly, cells were grown in 1x Dulbecco’s Modified Eagle’s medium (DMEM) with 10% fetal bovine serum (FBS), 25 mM glucose and 1% penicillin/streptomycin and maintained at 37°C with 5% CO_2_ in 12 well plates. Prior to incubation cells were serum starved for 16 hours overnight in DMEM minus neutral red.

### Cyclic AMP determination

Cyclic AMP levels in mHippoE-18 cells were determined as described previously in detail [20–23], except 100 μM isobutylmethylxanthine (IBMX) was used. Briefly, cells were pre-incubated with 100 μM IBMX for 20 min, followed by incubation with 10 μM forskolin and 100 μM IBMX in the presence of increasing concentrations of the various agonists for a further 20 min. In experiments where antagonists were used mHippoE-18 cells were pre-incubated with 100 μM IBMX and 1 μM of antagonist for 20 min, followed by incubation with varying concentrations of agonist, 1 μM antagonist, 10 μM forskolin and 100 μM IBMX for a further 20 min. Cyclic AMP levels were measured using a [^3^H]-cyclic AMP (NET275, Perkin Elmer) protein kinase A radiometric binding assay [24].

Cyclic AMP levels are represented as a percentage of basal samples unless otherwise stated. Student’s T-test (two-tailed and unpaired) was used to test for significance. Unless otherwise stated, all data are shown as mean ± SEM. Each data point plotted was the mean of data obtained from at least three experiments. Within each experiment three separate replicate wells were analysed for each condition and the cyclic AMP assays on each of the wells was carried out in duplicate.

Forskolin was used both to increase basal cyclic AMP levels to make it easier to detect increases and decreases in cyclic AMP levels in the same experiments and also to potentiate responses to agonists to more accurately determine their threshold effects [25]. A non-saturating 10 μM concentration of forskolin was used. Basal levels of mHippoE-18 cell cyclic AMP were (5.1 ± 0.42 pmoles / mg protein (n = 12)) and these were raised to (160.9 ± 7.2 pmoles / mg protein (n = 123)) after exposure to 10 μM forskolin. Protein levels were determined using a Bradford assay.

### Phospho-ERK determination

Phospho-extracellular signal-related kinase (Phospho-ERK) levels were determined essentially as described previously [22, 26–28]. Minor modifications have been described [20, 21]. ERK1/2 phosphorylation levels were quantified by densitometry. The developed films were scanned in and analysed using the software program, Aida. The data generated by Aida was then processed using Microsoft Excel. Values for the kinase expression and activity levels were defined as 100% in the control samples. Student’s T-test (two-tailed and unpaired) was used to test for significance.

### Drugs

The drugs used in these experiments were obtained from the following sources:

17β-Estradiol, aldosterone and IBMX were purchased from Sigma-Aldrich (Poole, Dorset, UK); G1, G15, G36, 2,3-*bis*(4-Hydroxyphenyl)-propionitrile (DPN), 4,4’,4”-(4-propyl-[1*H*]-pyrazole-1,3,5-triyl)*tris*phenol (PPT), 7α, 17β-[9-[(4,4,5,5,5-Pentafluoropentyl)sulfinyl]nonyl]estra-1,3,5(10)-triene-3,17-diol (ICI 182,780) and (2)-2-[4-(1,2-Diphenyl-1-butenyl)phenoxy]-*N*,*N*-dimethylethanamine citrate (Tamoxifen citrate) were purchased from Tocris Bioscience (Bristol, UK). Forskolin was obtained from Abcam Biochemicals (Cambridge, UK). We thank Professor Jeffrey Arterburn, New Mexico State University, Las Cruces, New Mexico, USA for initial samples of G36.

## Results

### Activation of adenylyl cyclase activity in mHippoE-18 cells

#### Agonist specificity

**Fig 1:**
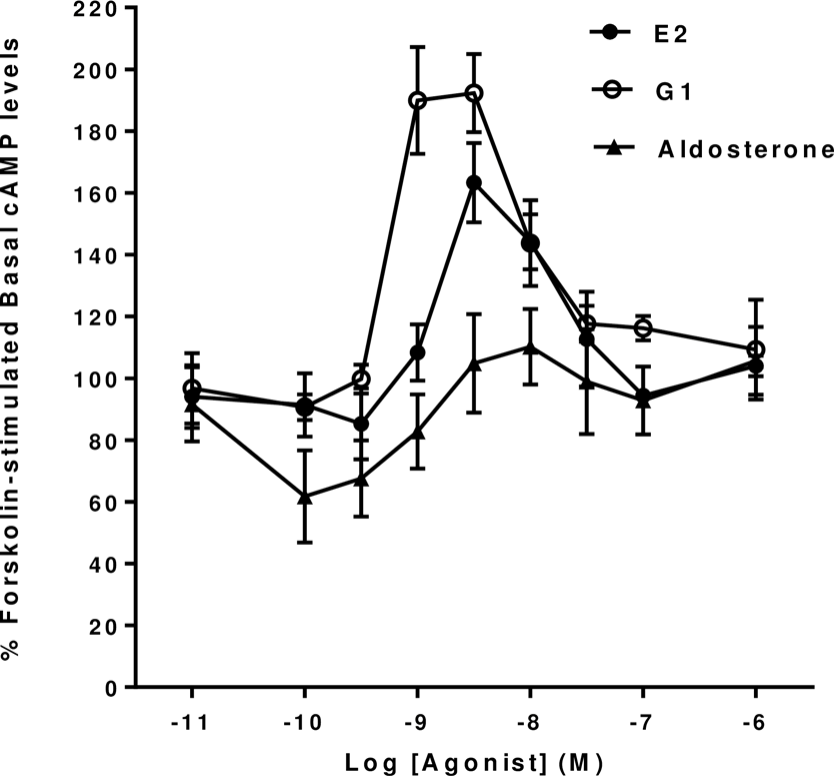
Effect of 17β-Estradiol, Aldosterone and G1 on basal forskolin-stimulated cAMP levels in mHippoE-18 cells. Cells were pre-incubated with 100 μM IBMX for 20 min, followed by incubation with 10 μM forskolin and 100 μM IBMX in the presence of increasing concentrations of the various agonists for a further 20 min. The basal level in the absence of agonist is shown as 100%. Values are significantly different from basal as follows: 17β-Estradiol, 3 nM p <0.001, 10 nM p <0.01; G1, 1 nM p <0.005, 3 nM p <0.001, 10 nM p <0.05; Aldosterone, 0.1 nM and 0.3 nM p < 0.01, 1 nM p <0.05. Data are expressed as the mean ± SEM. *n* ≥ 3.

GPER1 is able to activate a wide range of second messenger pathways in different cell types (see [1, 2]). However, since it appears to be able to regulate intracellular cyclic AMP levels in hippocampal and other neuronal cells, we initially investigated whether it was able to produce similar effects in mHippoE-18 cells when activated by a number of potential agonists. It can be seen that the application of both 17β-Estradiol and the GPER1 agonist, G1, produced dose-dependent increases in forskolin-stimulated cyclic AMP levels in mHippoE-18 cells (Fig 1). G1 showed a lower threshold than 17β-Estradiol for an increase in cyclic AMP levels together with an increased maximal response. G1 appeared to be slightly more potent than 17β-Estradiol with EC_50_’s of 5.2 x 10^−10^ M and 1.1 x 10^−9^ M respectively. The responses to both compounds declined at all concentrations tested above 3 x 10^−9^ M.

The mineralocorticoid, aldosterone, has also been suggested to be able to activate GPER1 in a number of different preparations from the cardiovascular system and other cell types [3, 29–31]. In mHippoE-18 cells aldosterone, at concentrations up to 10^−6^ M, did not produce any significant increases in cyclic AMP levels at any of the concentration tested. However, it did produce a significant decrease in forskolin-stimulated cyclic AMP levels in these cells at concentrations between 10^−10^ M and 10^−9^ M.

**Fig 2:**
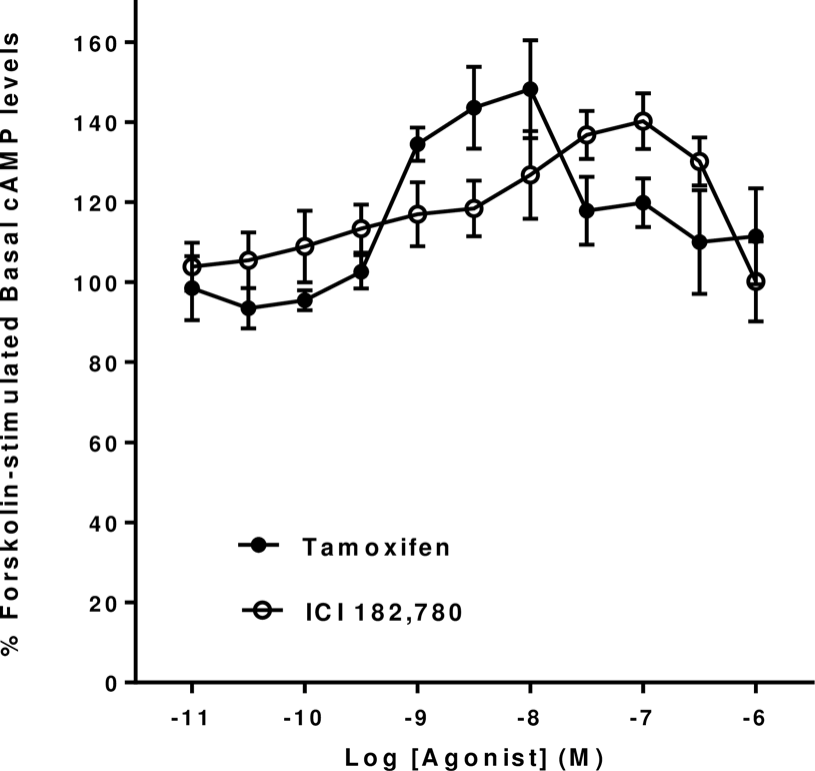
Effect of Tamoxifen and ICI 182,780 on basal forskolin-stimulated cAMP levels in mHippoE-18 cells. Cells were pre-incubated with 100 μM IBMX for 20 min, followed by incubation with 10 μM forskolin and 100 μM IBMX in the presence of increasing concentrations of the various agonists for a further 20 min. The basal level in the absence of agonist is shown as 100%. Values are significantly different from basal as follows: Tamoxifen, 1 nM p <0.01, 3 nM p <0.005, 10 nM p <0.005; ICI 182,780, 3 nM p <0.05, 10 nM p <0.05, 30 nM p <0.01, 100 nM p <0.005, 300 nM p <0.05. Data are expressed as the mean ± SEM. *n* ≥ 3.

In many preparations GPER1 responses have been shown to be induced by drugs which have traditionally been shown to be antagonists of the classical estrogen receptors, ER*α* and ER*β* [1, 5]. Thus, we examined the effects of Tamoxifen and ICI 182,780 on forskolin-stimulated cyclic AMP levels in mHippoE-18 cells. It can be seen that both compounds produced dose-dependent increases in cyclic AMP levels in the mHippoE-18 cells (Fig 2). Tamoxifen showed a lower threshold for activation of a significant response (between 3 x 10^−10^ M and 10^−9^ M) than ICI 182,780 (between 10^−9^ M and 3 x 10^−9^ M). Tamoxifen was also slightly more potent than ICI 182,780 with EC_50_’s of 6.4 x 10^−10^ M and 3.6 x 10^−9^ M respectively.

**Fig 3:**
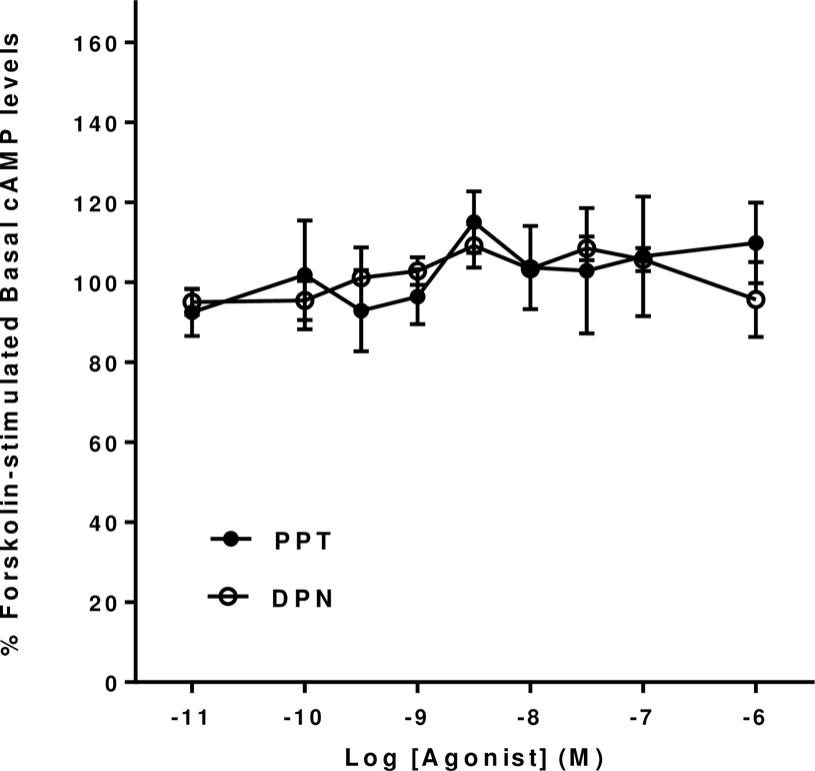
Effect of PPT and DPN on basal forskolin-stimulated cAMP levels in mHippoE-18 cells. Cells were pre-incubated with 100 μM IBMX for 20 min, followed by incubation with 10 μM forskolin and 100 μM IBMX in the presence of increasing concentrations of the various agonists for a further 20 min. The basal level in the absence of agonist is shown as 100%. Data are expressed as the mean ± SEM. *n* ≥ 3.

The nuclear ERα agonist, PPT, and the nuclear ERβ agonist, DPN, did not show any significant increases or decreases in forskolin-stimulated cyclic AMP levels in mHippoE-18 cells at concentrations up to 10^−6^ M (Fig 3).

#### Effect of antagonists

**Fig 4:**
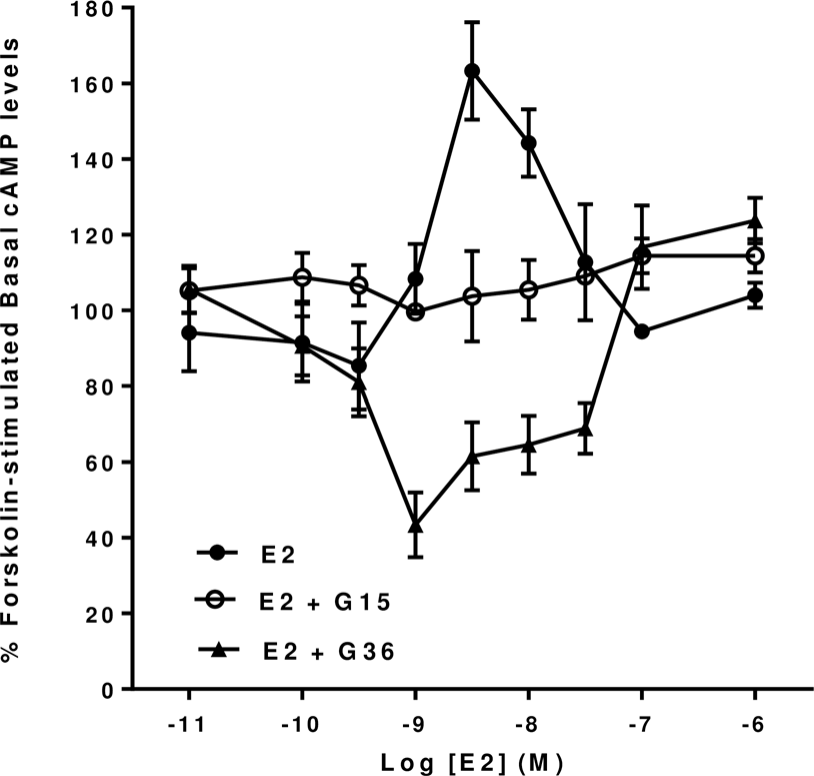
Effect of the GPER antagonists G15 and G16 on 17β-Estradiol (E2) forskolin-stimulated cAMP levels in mHippoE-18 cells. mHippoE-18 cells were pre-incubated with 100 μM IBMX and 1 μM of either of the antagonists for 20 min, followed by incubation with varying concentrations of 17β-Estradiol, 1 μM antagonist, 10 μM forskolin and 100 μM IBMX for a further 20 min. The basal value in the absence of agonist and antagonists is shown as 100%. The 17β-Estradiol-only response in the absence of antagonist is shown for comparison. Values are significantly different from basal as follows: E2, see Fig 1; E2 +G36, 1 nM p <0.001, 3 nM, 10 nM and 30 nM p <0.01. Data are expressed as the mean ± SEM. *n* ≥ 3.

The compounds G15 [32] and G36 [33] have been suggested to be specific antagonists of the effects of 17β-Estradiol and G1 on GPER1 mediated responses in a wide variety of clonal cell lines expressing GPER1 and also in a number of tissue preparations expressing GPER1. Thus, we have examined the effects of G15 and G36 on the forskolin-stimulated increases in cyclic AMP in mHippoE-18 cells induced by both 17β-Estradiol and by G1 (see above). It can be seen that G15, at a concentration of 10^−6^ M, completely blocks the dose-dependent 17β-Estradiol mediated increases in forskolin-stimulated cyclic AMP levels (Fig 4). Further, this response was also blocked by G36 at a concentration of 10^−6^ M (Fig 4). However, in the presence of 10^−6^ M G36, 17β-Estradiol revealed an additional significant dose-dependent inhibition of forskolin-stimulated cyclic AMP levels between 10^-9^M and 3 x 10^-8^M. In contrast, in the presence of 10^−6^ M G15, 17β-Estradiol appeared to slightly reduce cyclic AMP levels but this effect was not statistically significant.

**Fig 5:**
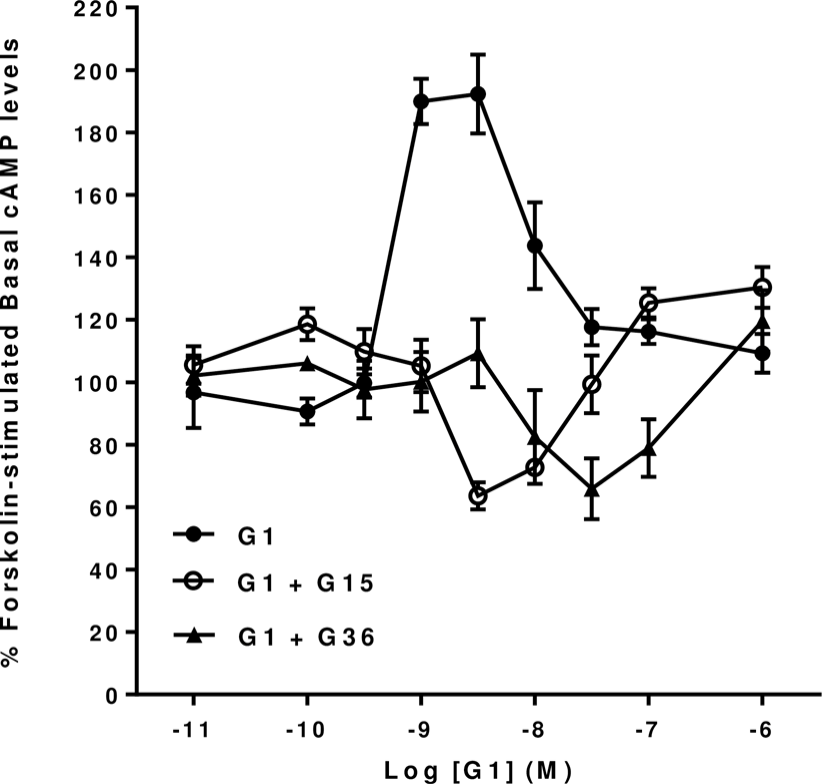
Effect of the GPER antagonists G15 and G16 on G1 forskolin-stimulated cAMP levels in mHippoE-18 cells. mHippoE-18 cells were pre-incubated with 100 μM IBMX and 1 μM of either of the antagonists for 20 min, followed by incubation with varying concentrations of G1, 1 μM antagonist, 10 μM forskolin and 100 μM IBMX for a further 20 min. The basal value in the absence of agonist and antagonists is shown as 100%. The G1-only response in the absence of antagonist is shown for comparison. Values are significantly different from basal as follows: G1, see Fig 1; G1 + G15, 3 nM p <0.01, 10 nM, p <0.05; G1 + G36, 10 nM and 100 nM, p <0.05, 30 nM p <0.01. Data are expressed as the mean ± SEM. *n* ≥ 3.

Similarly, the dose-dependent effects of G1 on forskolin-stimulated cyclic AMP levels in mHippoE-18 cells were also blocked by both G15 (10^−6^ M) and G36 (10^−6^ M) (Fig 5). However, G1 was able to produce a dose-dependent inhibition of forskolin-stimulated cyclic AMP levels, with similar maximal effects, in the presence of either G15 or G36. The inhibitory effect of G1 showed a threshold of between 10^−9^ M and 3 x 10^−9^ M in the presence of G15 and a significant effect at concentrations of 3 x 10^−9^ M and 10^−8^ M. Nonetheless, at higher concentrations (10^−7^ M and 10^−6^ M) G1 still showed a significant increase in cyclic AMP levels even in the presence of G15 (10^−6^ M). In contrast, the inhibitory effect of G1 in the presence of G36 showed a threshold effect only between 3 x 10^−9^ M and 10^−8^ M. The inhibitory effect of G1 appeared to be almost an order of magnitude more potent in the presence of G15 than in the presence of G36.

**Fig 6:**
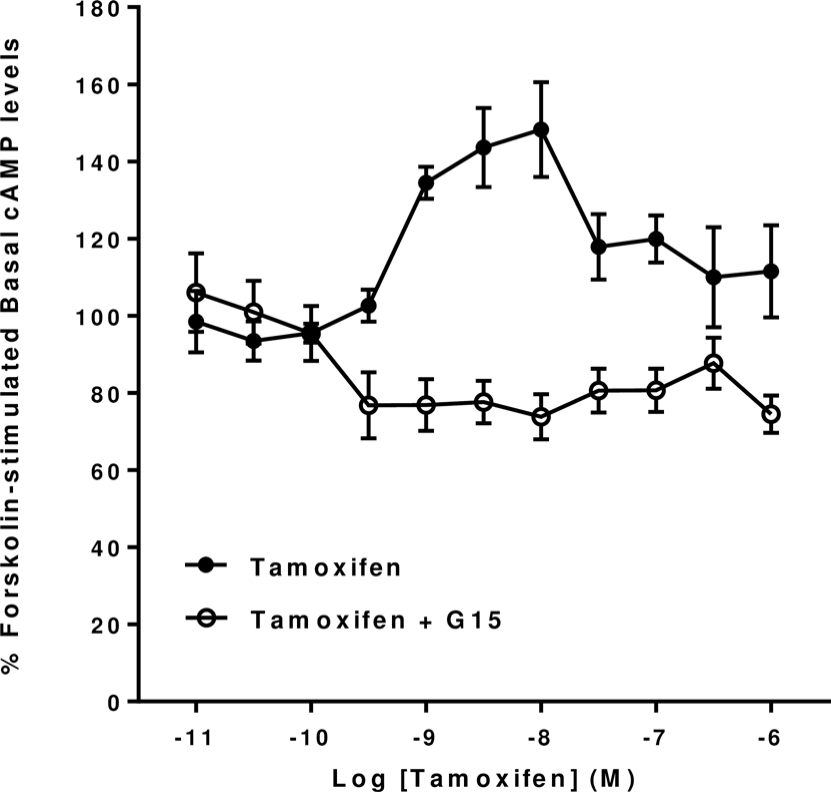
Effect of the GPER antagonist G15 on Tamoxifen forskolin-stimulated cAMP levels in mHippoE-18 cells. mHippoE-18 cells were pre-incubated with 100 μM IBMX and 1 μM of G15 for 20 min, followed by incubation with varying concentrations of Tamoxifen, 1 μM antagonist, 10 μM forskolin and 100 μM IBMX for a further 20 min. The basal value in the absence of agonist and antagonist is shown as 100%. The Tamoxifen-only response in the absence of antagonist is shown for comparison. Values are significantly different from basal as follows: Tamoxifen see Fig 2; Tamoxifen + G15, 0.3 nM and above (except for 300 nM), p <0.05. Data are expressed as the mean ± SEM. *n* ≥ 3.

**Fig 7:**
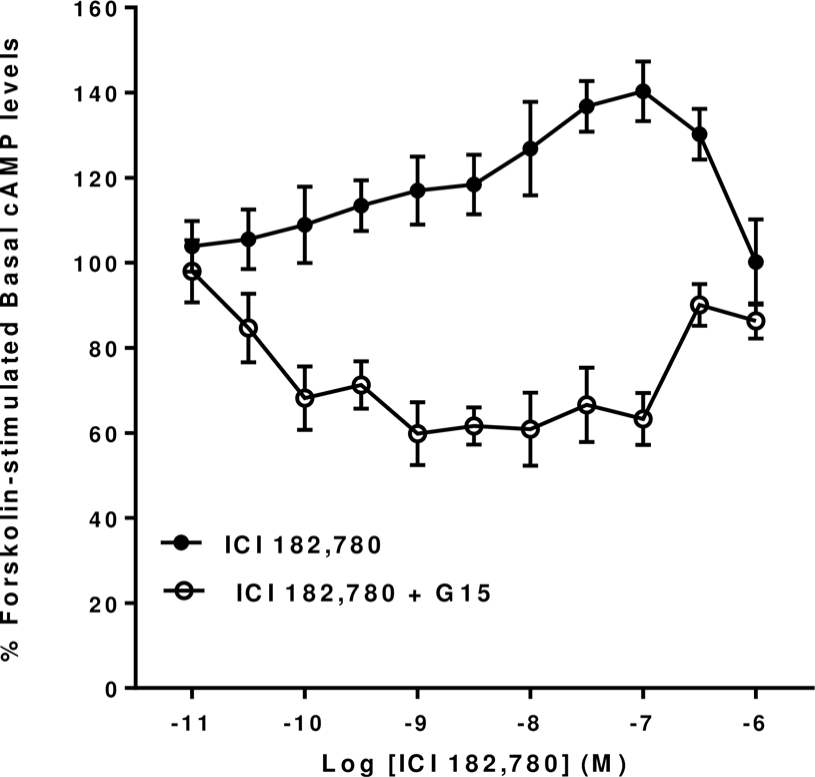
Effect of the GPER antagonist G15 on ICI 182,780 forskolin-stimulated cAMP levels in mHippoE-18 cells. mHippoE-18 cells were pre-incubated with 100 μM IBMX and 1 μM of G15 for 20 min, followed by incubation with varying concentrations of ICI 182,780, 1 μM antagonist, 10 μM forskolin and 100 μM IBMX for a further 20 min. The basal value in the absence of agonist and antagonist is shown as 100%. The ICI 182,780-only response in the absence of antagonist is shown for comparison. Values are significantly different from basal as follows: ICI 182,780 see Fig 2; ICI 182,780, 0.1 nM to 100 nM p <0.01. Data are expressed as the mean ± SEM. *n* ≥ 3.

To investigate further the unusual effects of Tamoxifen and ICI 182,780 on forskolin-stimulated cyclic AMP levels in mHippoE-18 cells we repeated them in the presence of G15 (10^−6^ M) to see if they were also likely to be mediated via GPER1. It can be seen that the dose-dependent stimulatory effects of both Tamoxifen and ICI 182,780 on cyclic AMP levels were both blocked by G15 (Figs 6 and 7). Further, they were both converted into dose-dependent inhibitory effects in the presence of G15. The inhibitory effects of ICI 182,780 had both a lower threshold and a greater magnitude that the corresponding effects produced by Tamoxifen.

### Activation of the mitogen-activated protein kinase (MAPK) pathway in mHippoE-18 cells

Since GPER1 activation has also been shown to be capable of coupling to the activation of the MAPK pathway in a number of different tissues (see [1]), we also investigated whether 17β-Estradiol and aldosterone exposure could increase the level of phosphorylation of ERK1/2 in mHippoE-18 cells using Western blotting. We exposed serum-starved mHippoE-18 cells to varying concentrations of 17β-Estradiol and aldosterone between 10^−8^ M and 10^−6^ M for varying times between 2 and 30 minutes. However, only extremely small and variable increases in the phosphorylation of ERK1/2 were obtained between 2 and 5 min of exposure to the agonists in contrast to highly significant responses to control applications of FBS to the mHippoE-18 cells (data not shown).

## Discussion

The present study has investigated the signalling properties of the estrogen activated G-protein coupled receptor (GPER1) in the immortalized hippocampal cell line, mHippoE-18. Application of 17β-Estradiol to the mHippoE-18 cells induces a rapid, and dose-dependent, increase in forskolin-stimulated cyclic AMP levels in these cells. The response is not mimicked by the application of the classical estrogen receptor agonists, PPT, (an ERα agonist) or DPN, (an ERβ agonist), suggesting that this effect of 17β-Estradiol may be mediated through the activation of GPER1. This suggestion is supported by the fact that the response is mimicked, in a dose dependent way, by the GPER1 agonist, G1 [34], and by the fact that both the stimulatory effects of 17β-Estradiol and of G1 on forskolin-stimulated cyclic AMP levels in the mHippoE-18 cells are inhibited by the antagonists G15 [32] and G36 [33] which are suggested to be specific for GPER1. In parallel with GPER1 mediated effects in a variety of other tissues [1], the classical ER antagonists, tamoxifen and ICI 182,780 acted as dose-dependent agonists on forskolin-stimulated cyclic AMP levels in the mHippoE-18 cells. Further, these agonist effects of tamoxifen and ICI 18720 were blocked in the presence of G15. Thus, the GPER1 receptor expressed in mHippoE-18 cells appears to exhibit a pharmacological profile similar to that observed for this receptor in a wide range of other cell types including other neuronal cell types and various types of cancer cells [1].

GPER1 has been shown to increase cyclic AMP levels by the Gs-mediated activation of adenylyl cyclase in a range of other tissues and when exogenously expressed in a range of clonal cell lines [4–6, 35]. Thus, GPER1 has been shown to increase cyclic AMP levels by the activation of adenylyl cyclase in human breast cancer cell lines, such as SKBR3, which lack ERα and ERβ [5] and in transfected human embryonic kidney 293 cells [36, 37]. In addition, GPER1 can increase levels of cyclic AMP by Gs activation of adenylyl cyclase in fish oocytes [38] and in vascular tissue [39]. However, in nervous tissue the role, and molecular mode of action, of GPER1 is not clear. GPER1 is widely expressed in many regions of the brain, including the hippocampus, the cortex and the hypothalamus (see [1]). In hippocampal cells it may mediate the protective effects of 17β-Estradiol on glutamate induced neurotoxicity [19] and on ischaemia [40]. GPER1 may also mediate the stimulatory actions of 17β-Estradiol on neuritogenesis in mouse primary hippocampal neurons via a pathway involving the PI3Kinase/AKt upregulation of neurogenin 3 [41]. In addition, GPER1 may also be involved in the control of the release of hormones from hypothalamic neurons (see [2, 42, 43]). The modulation of dendritic spines by 17β-Estradiol, which is thought to underlie some aspects of synaptic plasticity and the modulation of cognitive function (see [2, 11, 12, 44]) may also be mediated by GPER1 since this receptor has been described to be located in synaptic spines where it would be ideally located to mediate specific changes in spine morphology [2, 8, 9]. Many of the modulatory effects of GPER1 in the nervous system have been reported to be due to the activation of additional Gi mediated pathways involving either the P13K/Akt or the ERK1/2 pathways (see [2, 12]). The suggested mechanism of the GPER1 stimulation of the ERK1/2 pathway is very complex and involves the transactivation of the Epidermal Growth Factor Receptor. This “convoluted intracellular pathway” has been most extensively investigated in studies on breast cancer cells (see [5]). Although acute 17β-Estradiol treatment activates the STAT3 and Akt pathways in mHippoE-18 cells [19], we were unable to demonstrate a significant 17β-Estradiol activation of the ERK1/2 pathway in these cells, suggesting that GPER1 is unlikely to signal via this pathway in these cells.

The results of the present study using the proposed specific GPER1 antagonists G15 and G36, indicate that 17β-Estradiol and G1 can modulate cyclic AMP levels in mHippoE-18 cells by multiple pathways. In the presence of 10^−6^ M G36 both 17β-Estradiol and G1 produce a dose-dependent inhibition of forskolin-stimulated cyclic AMP levels in these cells. Similarly, in the presence of G15, G1, Tamoxifen and ICI 182,780 also produce a dose-dependent inhibition of forskolin-stimulated cyclic AMP levels in these cells. It is not clear why 17β-Estradiol did not produce a similar inhibition of this response in the presence of G15 but it did in the presence of G36. This may reflect differences in the properties of the GPER1 conformations induced by exposure to G15 and to G36.

At the present time the molecular mechanisms underlying this novel inhibition of forskolin-stimulated cyclic AMP levels in mHippoE-18 cells are not known. There would seem to be at least two possible general mechanisms. First, the inhibition of the forskolin-stimulated cyclic AMP levels by G15 and G36 could reveal the presence of a second receptor activated by 17β-Estradiol, G1, Tamoxifen and ICI 182,780, which is possibly coupled to a Gi-mediated inhibition of forskolin-stimulated cyclic AMP levels, the effects of which are normally masked by the stimulation of cyclic AMP levels by the activity of GPER1. The presence of such a second Gi-coupled receptor might also explain why, at high concentrations in the absence of the antagonists, G1 has a larger apparent efficacy in increasing cyclic AMP levels than 17β-Estradiol. If 17β-Estradiol had a larger efficacy for this second receptor than G1, its effects could be inhibited more if both receptors are activated at the same time. However, this could simply be because G1 has a higher efficacy than 17β-Estradiol at GPER1 in this preparation. Previous studies have reported the coupling of a range of GPCRs, such as the β2-adrenergic receptor [45] and the α2A-adrenergic receptor [46–48], to both the stimulation and inhibition of forskolin-stimulated cyclic AMP levels, under both concentration dependent and time dependent conditions. The classical estrogen receptors, ERα and ERβ have been shown to be expressed at moderate levels in mHippoE-18 cells [19] and they have been suggested to be localized to the plasma membranes of some cell types and may mediate some of the rapid non-genomic actions of 17β-Estradiol [12]. However, there do not appear to be any reports of these receptors underlying the inhibition of cyclic AMP levels in cells. In addition, the pharmacology of the inhibitory response, which demonstrates an activation by 17β-Estradiol, G1, Tamoxifen and ICI 182,780, seems to be very different from the pharmacological profiles reported for ERα and ERβ [1]. A number of additional membrane located receptors sensitive to 17β-Estradiol have been reported in the literature including a receptor sensitive to STX (a molecule which resembles 4-hydroxytamoxifen, the active component of tamoxifen) which couples to Gq (Gq-mER) [49]. It has been suggested that STX can also activate GPER1, as well as this additional receptor (see [1]). A further uncharacterized 17β-Estradiol sensitive receptor, ERX, has also been reported [14, 50]. However, the identity of the latter two receptors is unknown.

A second possibility would be that GPER1 adopts different conformations in the presence of so called biased ligands [51, 52]. Thus, the receptor conformations induced by 17β-Estradiol and G1, in the absence of the biased antagonists G15 and G36, could be different from those induced in the presence of the biased antagonists. The different receptor conformations could express differences in their abilities to couple to different second messenger pathways. There is much current interest in the concept of “Biased Agonism” or “Agonist-specific coupling” [53, 54] and this has been extended to studies on “Biased Antagonists” in recent years [55, 56]. The fact that the inhibition of forskolin-stimulated cyclic AMP levels in mHippoE-18 cells in the presence of G15 or G36 is induced by 17β-Estradiol, G1, tamoxifen and ICI 182,780 suggests that the receptor mediating this response also has a pharmacological profile similar to the actions of GPER1 in many tissues. The only difference is that it is not blocked in the presence of G15 and G36. It is interesting to note that GPER1, expressed in hippocampal cells, has been suggested to be constitutively linked to the inhibition of cyclic AMP levels via its interactions with membrane-associated guanylate kinases (MAGUKs), such as PSD-95 and SAP97, and with protein kinase A-anchoring protein 5 (AKAP5) [10]. However, this seems unlikely to be the explanation for the inhibition of forskolin-stimulated cyclic AMP levels in mHippoE-18 cells seen in the present investigation since the AKAP mediated pathway was reported to be insensitive to ligands such as 17β-Estradiol [10]. Further experimentation will be needed to determine the molecular mechanisms of the inhibition of forskolin-stimulated cyclic AMP levels in mHippoE-18 cells and the possible involvement of Gi- and AKAP-proteins.

The fact that aldosterone alone can specifically mimick the actions of 17β-Estradiol and G1 in the presence of G36 on the inhibition of forskolin-stimulated cyclic AMP levels in mHippoE-18 cells, but does not mimick their actions on the potentiation of forskolin-stimulated cyclic AMP levels in mHippoE-18 cells, is interesting in view of the current controversy surrounding the possible actions of aldosterone on GPER1 (see [3]). Aldosterone has been suggested to be a novel endogenous agonist for GPER1 in cardiovascular tissue [29–31] and has also been reported to couple GPER1 to the activation of the MAPKinase pathway when heterologously expressed in HEK293 cells [57]. However, it has not been possible to demonstrate aldosterone binding to GPER1 receptors when heterologously expressed in HEK293 cells or endogenously expressed in SKBR3 breast cancer cells or whole mouse renal tissue plasma membrane preparations [58].

Thus, mHippoE-18 cells [19] can provide an important resource to investigate the modulation of hippocampal cells by estrogen which does not suffer from the limitations of primary hippocampal cell cultures. They can also provide a more natural signalling environment than that provided by the exogenous expression of GPER1 receptors in clonal cell lines, in which to explore the influence of Agonist-Specific Coupling or Biased Agonism on the pharmacological properties of GPER1 [2, 57].
